# Treatment of advanced lung adenocarcinoma with EGFR L833V/H835L compound mutations using furmonertinib: two case reports and literature review

**DOI:** 10.3389/fmed.2025.1658583

**Published:** 2025-09-02

**Authors:** Shize Wang, Guoliang Shi, Qingyi Liu, Guangjie Liu, Yanjie Liu, Yaqing Han, Maogang Gao, Kaihong Han, Shaonan Xie

**Affiliations:** Department of Thoracic Surgery, The Fourth Hospital of Hebei Medical University (Hebei Tumor Hospital), Shijiazhuang, Hebei, China

**Keywords:** lung adenocarcinoma, EGFR mutation, p.L833V/p.H835L compound mutation, furmonertinib, case report

## Abstract

**Background:**

Lung cancer remains the leading cause of cancer-related mortality worldwide. With advancements in molecularly targeted therapies, Epidermal growth factor receptor (EGFR) mutations have emerged as critical therapeutic targets in advanced lung adenocarcinoma. However, the EGFR p.L833V/p.H835L compound mutations are relatively uncommon, and their clinical characteristics and therapeutic response to EGFR tyrosine kinase inhibitors (TKIs) remain poorly defined.

**Case presentation:**

This study reports two cases of advanced lung adenocarcinoma harboring the EGFR p.L833V/p.H835L compound mutation, both treated with furmonertinib. Case 1 was a 62-year-old male, and Case 2 was a 61-year-old female, both diagnosed through tissue biopsy and next-generation sequencing (NGS). Following treatment, both patients achieved partial response (PR), with progression-free survival (PFS) of 29 months and not available (NA, >12 months), respectively, demonstrating good tolerability.

**Results:**

Furmonertinib appears to be an effective treatment for advanced lung adenocarcinoma with EGFR p.L833V/p.H835L compound mutations.

**Conclusion:**

This study further supports the therapeutic potential of furmonertinib in EGFR-mutant lung cancer.

## Introduction

Lung cancer is the leading cause of cancer-related mortality worldwide. Epidermal growth factor receptor (EGFR) is the most common genetic alteration in lung adenocarcinoma, accounting for approximately 60% of cases in Asian patients (1). Common sensitizing mutations (e.g., exon 19 deletions, L858R) typically respond well to first-to third-generation EGFR tyrosine kinase inhibitors (EGFR-TKIs). In contrast, rare EGFR mutations account for approximately 10–15% of all EGFR mutations, with an even lower prevalence observed in Asian populations (approximately 4.4%) (2). The EGFR p.L833V/p.H835L compound mutation is exceedingly rare, with an estimated incidence of less than 1%, and its biological behavior and treatment strategies lack evidence-based support. This article presents two cases of advanced lung adenocarcinoma successfully treated with furmonertinib, aiming to provide clinical insights for this rare patient population and explore the therapeutic potential of EGFR-TKIs in compound mutations.

## Case reports

### Case 1

**Figure 1:**
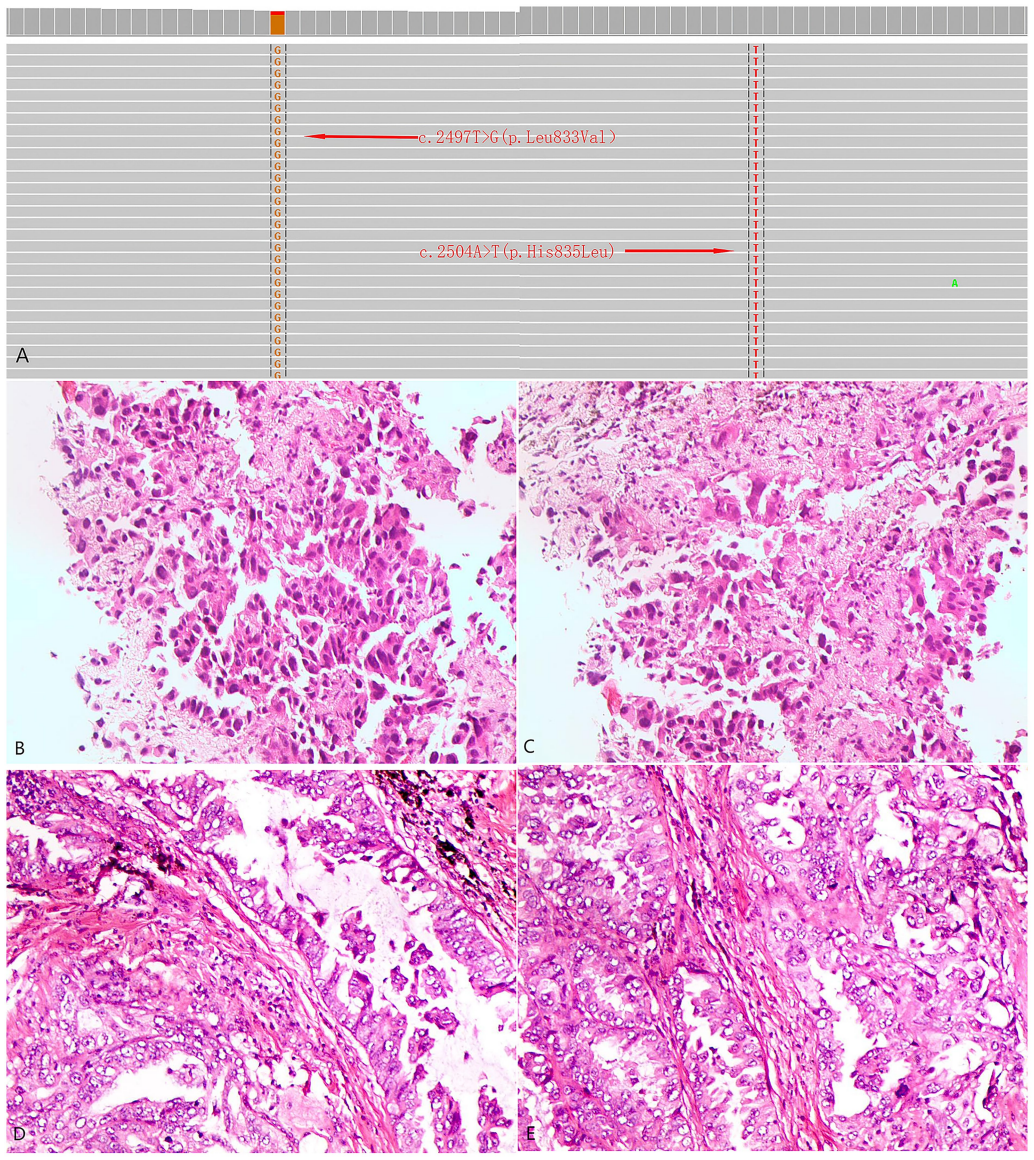
Next-generation genetic sequencing and histopathological profiles of the two patients. **(A)** Identification of L833V and H835L in EGFR exon 21 by next-generation sequencing. **(B,C)** Case 1: Histological sections stained with HE at a magnification of ×400 from a biopsy of a pulmonary mass. **(D,E)** Case 2: Histological sections stained with HE at a magnification of ×400 from a biopsy of a pulmonary mass.

**Figure 2:**
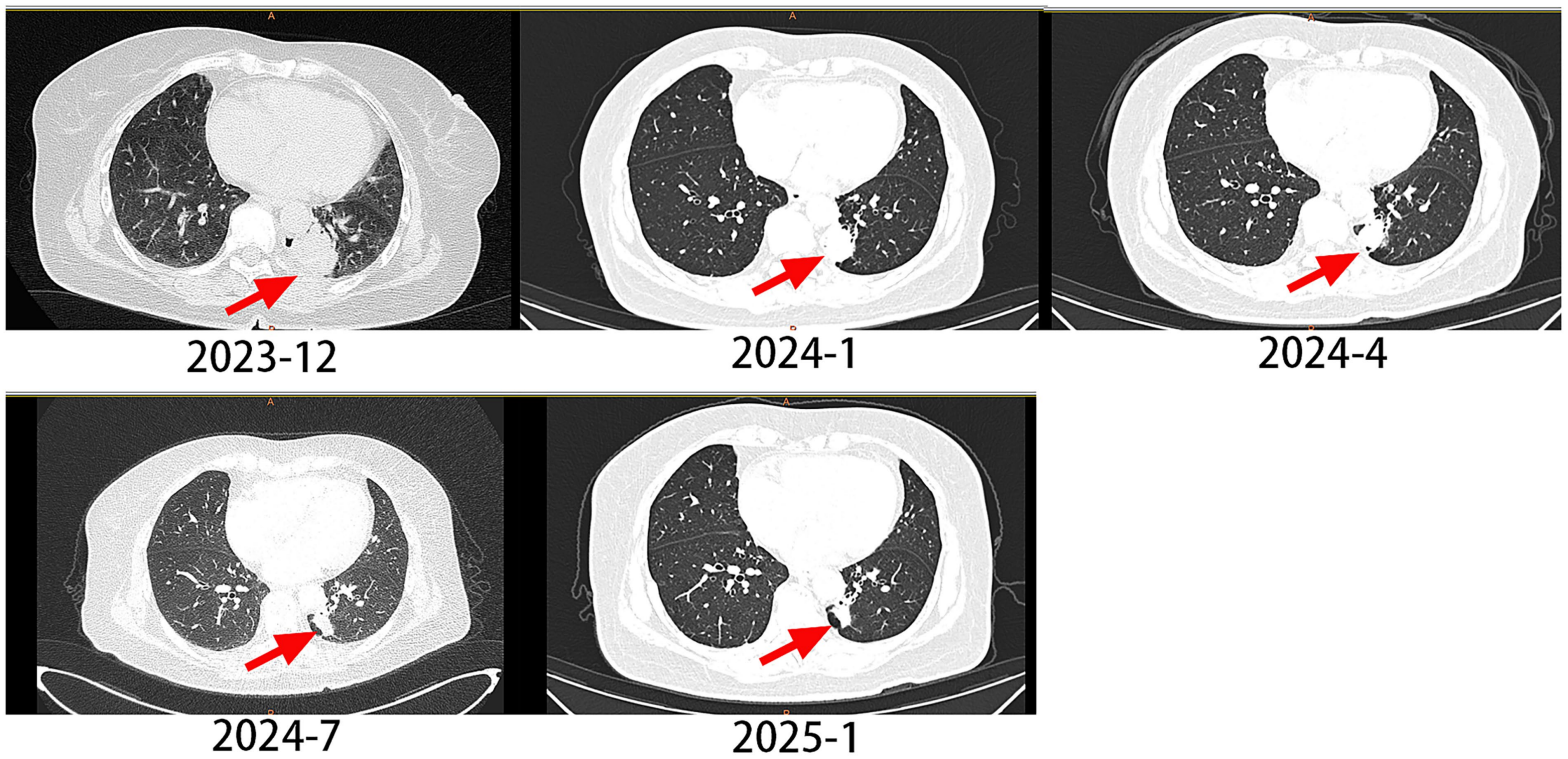
The changes of lung CT scan in case 1.

A 62-year-old male with a 20-year smoking history (quit 10 days prior to presentation) presented in December 2023 with cough and chest pain. Contrast-enhanced CT demonstrated a spiculated pulmonary mass measuring 3.9 × 3.1 cm in maximum cross-sectional diameter, exhibiting lobulated borders and pleural indentation in the left lower lobe, along with multiple bone metastases. The clinical stage was determined as cT2aN0M1c1, stage IVB. Pathology confirmed lung adenocarcinoma, and NGS testing detected an EGFR p.L833V/p.H835L compound mutation (Figure 1). The patient began oral furmonertinib (80 mg once daily) in January 2024. Follow-up CT scan at 1 month later showed the tumor size was 2.9 × 2.1 cm. Follow-up CT scans at 3, 6, and 12 months later revealed a progressive reduction in the lesion, with the size being 2.2 × 1.2 cm. According to the RECIST 1.1 criteria, the longest diameter of the tumor was reduced by ≥30%, and the efficacy was evaluated as PR (partial response) (Figure 2). The PFS exceeded 12 months, and no related adverse reactions occurred during the oral administration period. As of the time of writing, the patient remains on furmonertinib therapy.

### Case 2

**Figure 3:**
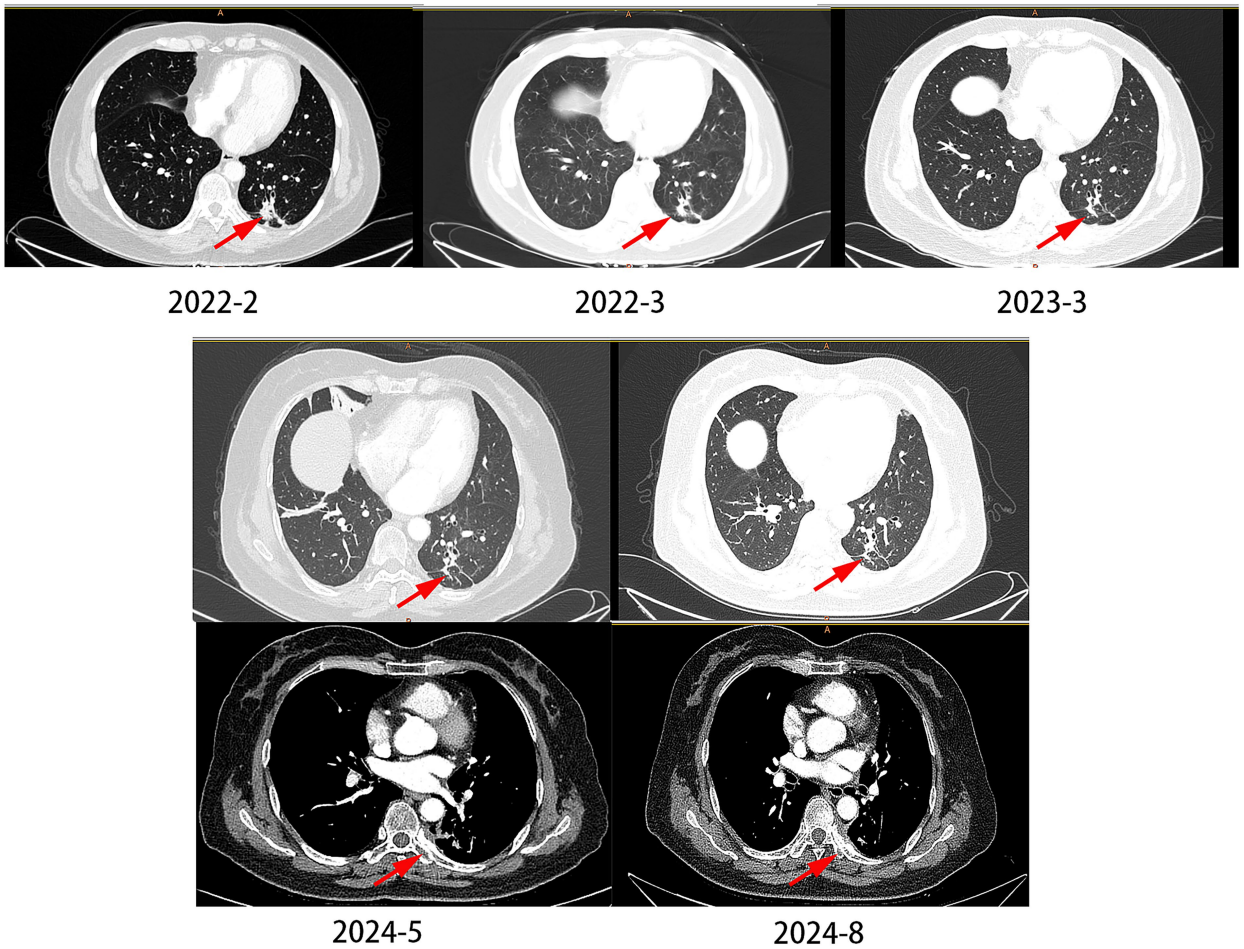
The changes of lung CT scan in case 2. In 2024–2028, the patient developed bone metastases, but the pulmonary nodules did not significantly enlarge.

The patient was a 61-year-old female, never-smoker, who presented with dyspnea in February 2022. Histopathological analysis of a biopsy confirmed adenocarcinoma (Figure 1), with no distant metastases detected at the time of diagnosis. The patient underwent surgical exploration in February 2022, during which widespread pleural metastases were observed. As a result, only an exploratory procedure was performed, without tumor resection. The tumor was staged as cT1cN0M1a, stage IVA. NGS testing identified an EGFR p.L833V/p.H835L compound mutation. The patient began oral furmonertinib (80 mg once daily) in March 2022. During the medication period, mild skin rash occurred (CTCAE grade 2), which improved after symptomatic treatment for 1 month. Follow-up CT scan at 1 month later showed tumor shrinkage, and subsequent CT re-examinations every 3 months documented continuous reduction of the tumor to 1.1 cm × 0.7 cm. Rib metastasis was detected in the re-examination in August 2024, with a PFS (progression-free survival, the time from the start of the study to the first occurrence of disease progression or death from any cause) of 29 months (Figure 3).

## Discussion

Lung cancer remains the leading cause of cancer-related mortality worldwide. EGFR represents the most frequent genetic alteration in lung adenocarcinoma, accounting for approximately 60% of cases in Asian populations (1). Although targeted therapy has significantly improved the prognosis of patients with advanced non-small cell lung cancer (NSCLC), the treatment of patients with rare EGFR compound mutations remains a challenge. Classic sensitizing mutations (e.g., exon 19 deletions and L858R) account for 85–90% of EGFR mutations and demonstrate favorable responses to first-and second-generation EGFR-TKIs, such as gefitinib (3) and afatinib (4) However, rare EGFR mutations account for approximately 10–15% of all EGFR alterations. These mutations may occur in isolation or coexist with common mutations or other rare variants as compound mutations, exhibiting striking heterogeneity and marked variability in sensitivity to EGFR-TKIs, with generally lower response rates compared to classic mutations (5, 6).

Third-generation EGFR-TKIs (e.g., osimertinib, furmonertinib) demonstrate therapeutic efficacy against certain rare mutations due to their broad-spectrum inhibitory activity. Floc’h N et al. (7) reported that osimertinib effectively suppressed signaling pathways and cellular proliferation in G719X-mutant cell lines *in vitro*. Their study further validated sustained tumor growth inhibition in patient-derived xenograft (PDX) models harboring G719X mutations either alone or in combination with L861Q and S768I. Zhang et al. (8) provided clinical evidence supporting osimertinib’s efficacy in NSCLC patients with rare EGFR mutations (including G719X, L861G, and S768I), achieving a mPFS of 8.2 months.

Furmonertinib, with its innovative trifluoroethoxypyridine structure, offers significant advantages in efficacy, safety, and coverage of uncommon mutations (9). Notably, clinical case reports have documented favorable outcomes (14 months of continuous treatment) in patients with rare G719X/S768I co-mutations treated with furmonertinib (10). This study is the first to report the significant efficacy of furmonertinib in treating EGFR p.L833V/p.H835L compound mutations. One patient achieved a PFS of 19 months, while the other had a PFS of NA (>12 months). These findings suggest that furmonertinib may overcome the efficacy limitations of traditional EGFR-TKIs in rare compound mutations, providing new evidence for clinical decision-making.

The EGFR p.L833V/p.H835L compound mutation is an extremely rare mutation, with only a few reported cases (11–25) (Table 1). We collected 15 previously reported cases with detailed clinical data, all of which involved lung adenocarcinoma patients with the EGFR p.L833V/p.H835L compound mutation, predominantly from Asian populations. The age of patients ranged from 36 to 89 years, with a male-to-female ratio of 11: 4. Most patients were in advanced stages, and all had received EGFR-TKI treatment.

**Table 1:** Clinical features and prognosis of NSCLC patients with EGFR p.L833V/p.H835L compound mutations.

Author/Report year	Age (y)/Gender	Race	Smoker	TNM	Metastases	Mutations	Treatment	PFS (months)
Yang et al. 2011 (11)	89/M	Asian	Yes	IV	Lung	p.L833V/p.H835L	Gefitinib (second-line)	8.5
Zhuang et al. 2013 (12)	48/M	Asian	No	IIB	NO	p.L833V/p.H835L	NA	NA
Frega et al. 2016 (13)	70/M	White	Yes	NA	NA	p.L833V/p.H835L/p.E709K	Afatinib	>2
Qin et al. 2018 (14)	36/M	Asian	No	IV	Pleural Bone Brain	p.L833V/p.H835L/p.R670W	Gefitinib (first-line) Afatinib (third-line) Osimertinib (fifth-line)	1 (Gefitinib), 7 (Afatinib), >3 (Osimertinib)
Li et al. 2019 (25)	77/F	Asian	No	IV	NA	p.L833V/p.H835L	Gefitinib	>15
Long et al. 2020 (15)	65/M	Asian	No	IV	NA	p.L833V/p.H835L	Afatinib	>10
Li et al. 2021 (18)	75/M	Asian	NO	IV	Brain	p.L833V/p.H835L	Afatinib (first-line) Osimertinib (second-line)	12 (Afatinib) NA (Osimertinib)
Yang et al. 2022 (16)	65/M	Asian	Yes	IV	Skin	p.L833V/p.H835L	Gefitinib	>18
Smith et al. 2023 (17)	60/F	NA	Yes	IV	Brain	p.L833V/p.H835L	Osimertinib	19
Miao et al. 2023 (19)	40/M	Asian	NA	IV	Lung Bone	p.L833V/p.H835L	Afatinib + Anlotinib	>5
Yang et al. 2023 (20)	44/F	Asian	NO	IV	Pleural Lung	p.L833V/p.H835L	Ectinib + chemotherapy	9
Luo et al. 2023 (21)	53/F	Asian	NO	IIIA	NO	p.L833V/p.H835L	Osimertinib	>22
Li et al. 2023 (23)	53/F	Asian	YES	IV	Brain	p.L833V/p.H835L	Almonertinib (second-line) Afatinib (third-line)	18 (Almonertinib) 0 (Afatinib)
Jowan et al. 2024 (22)	75/F	White	NA	IV	Brain	p.L833V/p.H835L	Osimertinib	>3
Pan et al. 2025 (24)	66/M	Asian	NO	IV	Pleural	p.L833V/p.H835L	Afatinib (first-line) Almonertinib (second-line)	24 (Afatinib) NA (Almonertinib)

Yang et al. (20) reported a 44-year-old Asian female patient with pleural effusion and bilateral lung metastases at diagnosis. Tumor shrinkage was observed after first-generation EGFR-TKI treatment, with a PFS of 9 months. Li et al. (18) described a 75-year-old Asian male patient with stage IV disease (TNM classification). After 14 months of second-generation TKI (afatinib) treatment, brain metastasis developed. A repeat biopsy with next-generation sequencing (NGS) revealed an acquired EGFR T790M resistance mutation. The patient was subsequently switched to a third-generation TKI with favorable therapeutic response. Another case reported by Li et al. (23) involved a 53-year-old Asian female patient with baseline brain metastases. Tumor progression occurred after 18 months of third-generation TKI (almonertinib) treatment. Switching to a second-generation TKI failed to demonstrate therapeutic efficacy.

We summarized the currently reported cases and found that, for the rare EGFR p.L833V/p.H835L compound mutation, third-generation TKIs show better efficacy than first-and second-generation TKIs. However, in Case 2, the patient developed bone metastases at the 29th month of oral furmonertinib administration. Due to the inconvenience of obtaining samples, we did not perform re-biopsy for genetic testing nor conduct testing via blood sampling. Among the three reported cases that upgraded their TKI therapy, resistance mutations such as T790M were detected. Additionally, in one case where the TKI treatment was downgraded, tumor progression was observed after switching therapy. It is highly likely that drug-resistant mutations, such as C797S, have also emerged in this patient.

In the two cases we reported, genetic testing confirmed the presence of the EGFR p.L833V/p.H835L compound mutation, and furmonertinib was chosen as the first-line treatment. Both patients exhibited remarkable clinical responses, making this the first documented report of furmonertinib as a first-line therapy for the rare EGFR p.L833V/p.H835L compound mutation.

## Conclusion

Furmonertinib appears to be an effective treatment for advanced lung adenocarcinoma with EGFR p.L833V/p.H835L compound mutations. This study further supports the therapeutic potential of furmonertinib in rare EGFR-mutant lung cancer. Additionally, we reviewed previously reported cases of non-small cell lung cancer (NSCLC) with the rare EGFR p.L833V/p.H835L compound mutation, summarizing their clinical characteristics and responses to different EGFR-TKIs. Despite the many challenges in the clinical management of rare EGFR mutations, substantial progress has been made in treatment strategies for this population in recent years. Moving forward, it is hoped that a global registry platform for rare EGFR mutations will be established, integrating multicenter clinical data with molecular characteristics to provide treatment experience for rare mutations.

## Data Availability

The original contributions presented in the study are included in the article/supplementary material, further inquiries can be directed to the corresponding author.
